# Crystal structure of ferric recombinant horseradish peroxidase

**DOI:** 10.1007/s00775-025-02103-2

**Published:** 2025-03-07

**Authors:** Mst Luthfun Nesa, Suman K. Mandal, Christine Toelzer, Diana Humer, Peter C. E. Moody, Imre Berger, Oliver Spadiut, Emma L. Raven

**Affiliations:** 1 School of Chemistry, University of Bristol, Bristol, UK; 2 School of Biochemistry, University of Bristol, Bristol, UK; 3 Institute of Chemical, Environmental and Bioscience Engineering, Research Division Biochemical Engineering, TU Wien, Vienna, Austria; 4 Leicester Institute for Structural & Chemical Biology, Department Molecular & Cell Biology, Henry Wellcome Building, University of Leicester, Leicester, LE1 9HN UK

**Keywords:** Horseradish peroxidase, Peroxidase, Ferulic acid, Heme

## Abstract

Horseradish peroxidase (HRP), isolated from horseradish roots, is heavily glycosylated, making it difficult to crystallize. In this work, we produced recombinant HRP in *E. coli* and obtained an X-ray structure of the ferric enzyme at 1.63 Å resolution. The structure shows that the recombinant HRP contains four disulphide bonds and two calcium ions, which are highly conserved in class III peroxidase enzymes. The heme active site contains histidine residues at the proximal (His 170) and distal (His 42) positions, and an active site arginine (Arg 38). Surprisingly, an ethylene glycol molecule was identified in the active site, forming hydrogen bonds with His 42 and Arg 38 at the δ-heme edge. The high yields obtained from the recombinant expression system, and the successful crystallization of the enzyme pave the way for new structural studies in the future.

**Graphical abstract:**

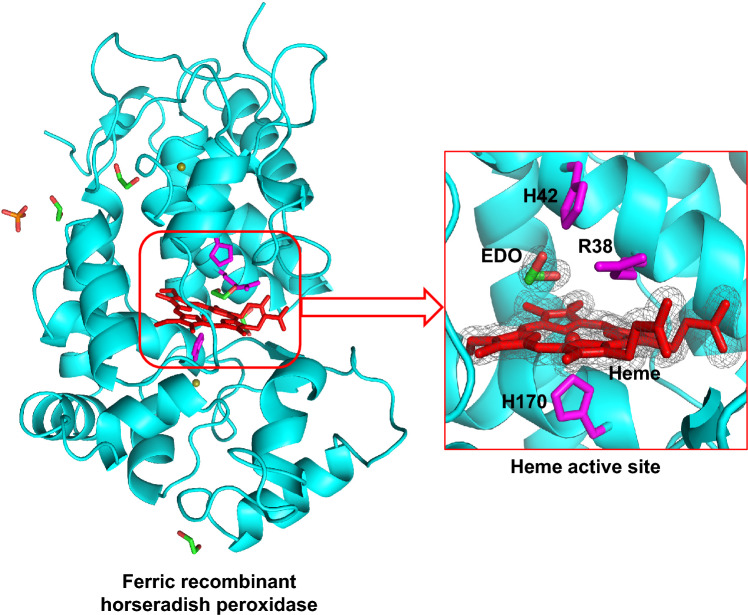

**Supplementary Information:**

The online version contains supplementary material available at 10.1007/s00775-025-02103-2.

## Introduction

Of all the heme peroxidases, horseradish peroxidase (HRP) is arguably the most iconic. Peroxidase activity was first identified in horseradish plants early in the nineteenth century [1]. But the native enzyme, as isolated from horseradish roots, is heavily glycosylated which has hampered its successful crystallization for decades. Almost 200 years after its enzymatic activity was first detected, HRP was eventually crystallized in recombinant form [2] and its structure solved [3]. Heme peroxidase enzymes are highly proficient in oxidizing small aromatic substrates, but HRP is the most proficient with catalytic activities that far outpace other peroxidases. Such high activity, producing easily detectable coloured or fluorescent products, makes HRP a very sensitive diagnostic tool in a wide range of biotechnological assays. That said, the difficulty of isolating a single isozyme of HRP from the many glycosylated isoenzymes present in its native source, alongside the extremely limited yields that are achieved from refolding of HRP from insoluble inclusion bodies in the recombinant *E. coli* expression system [4], means that only very few crystal structures of HRP have ever been published. 

Recently, an improved process for refolding of recombinant HRP from inclusion bodies of *E. coli* has been developed [5]. This produces pure, recombinant, unglycosylated HRP in yields that are more viable for biotechnological applications and in principle would allow more ambitious crystallography than has previously been possible. In this work, we have used this refolded recombinant HRP expressed from *E. coli* to crystallize ferric recombinant HRP and to determine its crystal structure.

## Methods

### Electronic spectra and steady state kinetics

Electronic spectra of both recombinant and commercially available (Sigma 77,332) HRP were measured in 10 mM phosphate buffer pH 7.0 using a Perkin Elmer Lambda 40 spectrophotometer. Steady-state kinetics of guaiacol oxidation were carried out at 25° C using HRP (12 nM), guaiacol (0.1 mM to 2.0 mM), and H_2_O_2_ (1.0 mM) in 10 mM phosphate buffer, pH 7.0, in a 500 µL cuvette according to published protocols [6].

### Crystallization of HRP, structure determination and refinement

HRP isozyme C1A was isolated from *Escherichia coli (E. coli)* using a multi-step inclusion body process, as previously described [5]. The expression system encodes for the HRP-C1A isozyme. Protein purity was assessed by SDS-PAGE (Figure S1) and from the R_z_ value (A_403_/A_280_ = 2.57).

Initially, ferric HRP was crystallized from previously reported conditions [2, 7] using a sitting drop vapour diffusion method at 4 °C. The crystallization drops were set up by mixing HRP (8.4 mg/ml) with saturated ferulic acid solution in isopropanol and with reservoir solution (20% polyethylene glycol (PEG) 8000, 0.2 M calcium acetate, 0.1 M sodium cacodylate, pH 6.5) in a ratio of 2:1:2 (HRP:ferulic acid:reservoir solution) using a Mosquito Xtal3 crystallization robot (SPT Labtech). Many small crystals (15–20 µm) were observed after 1 week.

To obtain better crystals, these initial small crystals were crushed using a seed-bead kit (Molecular Dimensions Ltd, UK) and used for micro-seeding in subsequent crystallization screens. In the next round of crystallizations, trays were set up using a sitting drop vapour diffusion at 4 °C with micro-seeding. The drops were set up in 196 (4x48) conditions available in Wizard Classic 1–4 (Molecular Dimensions Ltd, UK). The drop composition was HRP (7.82 mg/ml), saturated ferulic acid solution in isopropanol, and crystallization solution (3:1, Wizard classic condition:seed stock solution) in a ratio of 2:1:2 (HRP:ferulic acid:crystallization solution). We observed crystals in multiple conditions within 10–12 days as listed in Table S1.

Crystals (approximate size 200 µm × 30 µm × 40 µm) were harvested from the drop corresponding to the condition 25% (w/v) PEG 1500, 100 mM SPG (2:7:7, succinic acid:sodium hydrogen phosphate:glycine buffer, pH 8.5). The crystals were prepared for X-ray diffraction by brief immersion in the reservoir solution containing 20% ethylene glycol for cryoprotection, followed by flash freezing in liquid nitrogen.

X-ray diffraction data were collected from Diamond Light Source on I24 MX beam line. 7200 images of 0.05 degree were collected with an exposure time of 0.005 s using 0.999 Å radiation. Data were processed with autoPROC (version 1.0.5) [8] using XDS (Build 20,230,630) [9], along with Pointless (version 1.12.15) [10] and Aimless (version 0.7.13) [11] software from CCP4 (version 8.0.016) program suite [12]. The structures were solved by molecular replacement [13] using the structure of HRP-C (PDB 1GW2) as the search model. The model building and refinement were carried out using *PHENIX* (version 1.21) [14] and *Coot* (version 0.9.8) [15]. Data collection and refinement statistics are given in Table 1. Crystals diffracted to 1.63 Å, belonged to the space group *P2*_*1*_*2*_*1*_*2*_*1*_, and contained one molecule in the asymmetric unit. The coordinates and diffraction data have been deposited under PDB 9H1M.

**Table 1:** Data collection and refinement statistics

Data collection	HRPC1A
Beamline	Diamond light source I24
Wavelength (Å)	0.9999
Resolution range (Å)	58.20–1.63 (1.66–1.63)^1^
Space group	*P2*_*1*_*2*_*1*_*2*_*1*_
* a, b, c* (Å)	40.16, 67.06, 117.18
* α, β, γ* (°)	90.00, 90.00, 90.00
Observations	
Total reflections	144,807 (3961)^1^
Unique reflections	37,103 (1850)^1^
Multiplicity	3.9 (2.1)^1^
Completeness (%)	92.0 (93.6)^1^
Mean I/σ	5.4 (0.8)^1^
Wilson B-factor	22.02
R_merge_	0.115 (1.187)^1^
CC (1/2)	0.991 (0.343)^1^
Refinement	
R_work_	0.1709
R_free_	0.1893
Number of nonhydrogen atoms	
Protein	2375
Ligand	70
Solvent	140
Root mean square deviations from ideal values	
Bond length (Å)	0.005
Bond angle (°)	0.876
Ramachandran plot	
Favoured	97.7%
Allowed	2.3%
Outliers	0.0%
ROTAMER	
Outliers (%)	0.75%
Clash score	1.04
Mean B-factor (Å)	27.30
Macromolecules	26.97
Ligand	26.17
Solvent	33.36

^*1*^*The values in the bracket refer to the statistics for the highest resolution shell*

## Results

**Fig. 1:**
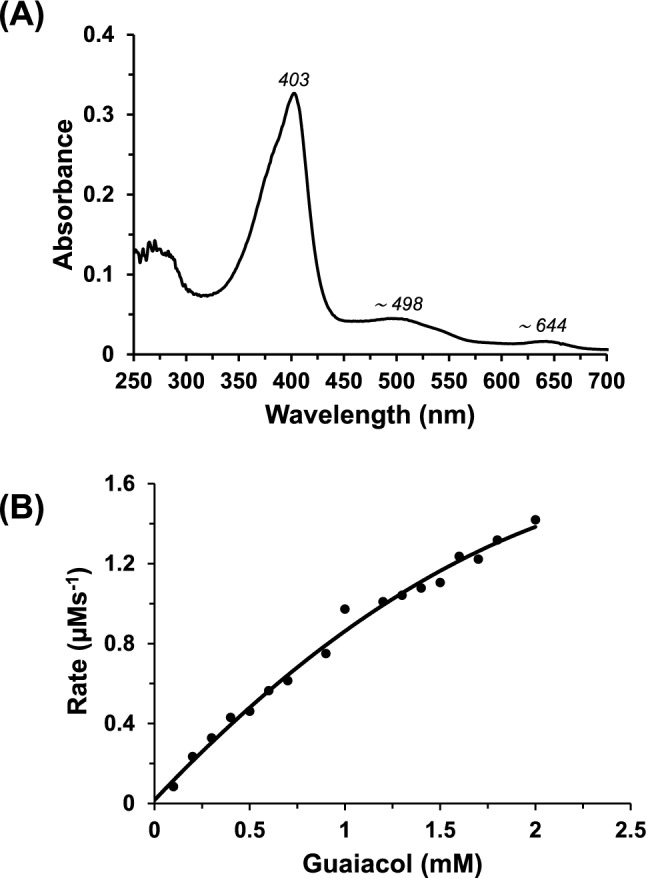
**A** The UV–visible spectrum of recombinant ferric HRP (10 mM phosphate buffer, pH 7.0, 25° C). Addition of ferulic acid leads to no substantive change in the ferric spectrum. **B** Steady-state oxidation of guaiacol by recombinant HRP. The solid line shows the fit of the data to the Michaelis–Menten equation ([HRP] = 12 nM, 10 mM phosphate buffer, pH 7.0, 25 °C)

The UV–visible spectrum of ferric recombinant HRP showed a Soret maxima at 403 nm (Fig. 1A), which is indicative of a ferric high-spin heme. This spectrum is consistent with those previously reported for the native wild-type HRP (403 nm [16, 17]) and other recombinant wild-type HRPs prepared using different approaches (402 nm [4, 18, 19]). Steady-state oxidation of guaiacol for recombinant HRP (*k*_cat_ = 304 ± 99 s^−1^ and *K*_m_ = 3.23 ± 1.50 mM, Fig. 1B) was in agreement with the values reported previously [20].

HRP crystals have previously been obtained under a very limited range of conditions (using cacodylate buffer in all cases, and ferulic acid as additive in most cases, Table S2). The availability of well-developed screening agents now means that different crystallization conditions can be explored, and several of these yield crystals that are potentially viable for X-ray work, Table S1.

**Fig. 2:**
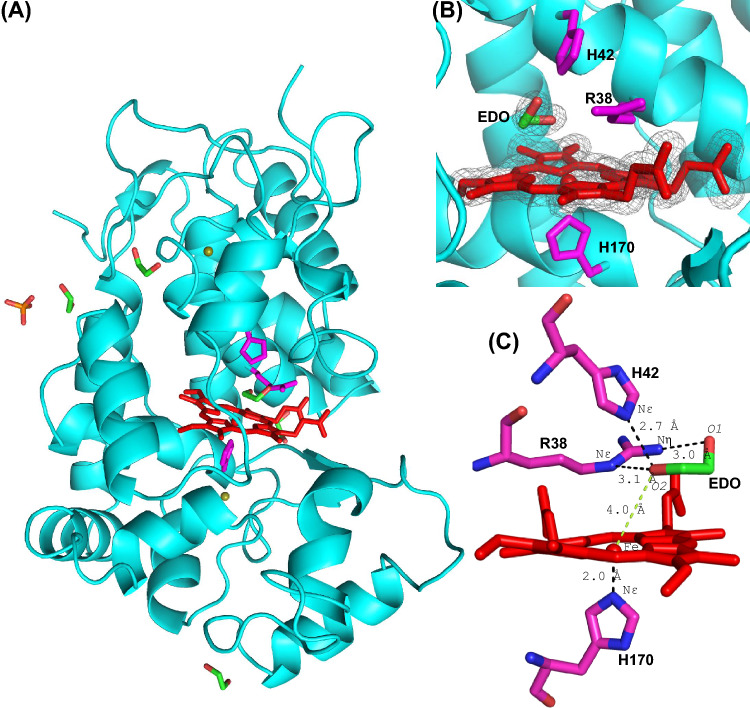
The structure of recombinant ferric HRP. **A** The overall structure of HRP, showing the heme (in red), the proximal, and distal histidine residues and arginine residue (His 170, His 42, and Arg 38, respectively, in magenta) and ethylene glycol (EDO, green), calcium ions (olive green) and phosphate (orange). **B** The active site, showing the heme, ethylene glycol, and the active site residues. Colour scheme is as in (**A**); electron density (*2F*_*o*_*-F*_*c*_) map of the heme and ethylene glycol in the active site, contoured at 1.3σ. **C** Ethylene glycol forms hydrogen bonds with His 42 and Arg 38 (black dotted lines) and is at 4.0 Å from the heme iron (green dotted line). Colour scheme is the same as in (**A**). The figure was produced using PyMol [37]

The crystal structure of HRP is shown in Fig. 2. The structure contains a total of 306 residues with 15 α-helices (148 residues) and 2 anti-parallel β-sheets (6 residues) (Fig. 2A). HRP contains four disulphide bonds (between residues C11–C91, C44–C49, C97–C301, and C177–C209), which are highly conserved among the class III peroxidases and observed in our structure. Additionally, there are two calcium ions present in the structure, although not in the vicinity of the active site. We note that there are four ethylene glycol molecules bound on the surface of HRP in the structure, along with one phosphate ion. The overall structure is in good agreement with other HRP structures (e.g. 0.192 Å all-atom RMSD with 6ATJ, 0.206 Å all-atom RMSD with 2ATJ).

**Fig. 3:**
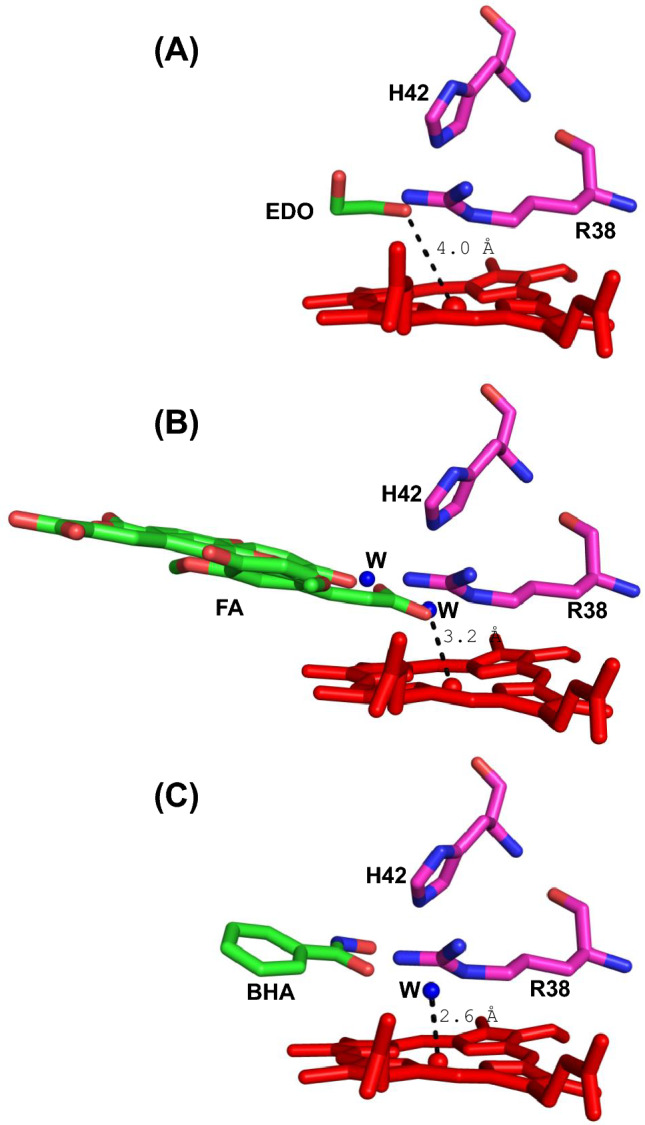
Comparison of binding locations in HRP. **A** Ethylene glycol (EDO, PDB ID 9H1M). **B** Ferulic acid (FA, PDB ID 6ATJ) [7]. **C** Benzhydroxamic acid (BHA, PDB ID 2ATJ) [22]. Colour scheme showing the heme (red), the Arg 38, and His 42 (magenta), and ethylene glycol, ferulic acid and benzhydroxamic acid (green). Water molecules are shown as blue spheres. The figure was produced using PyMol [37]. Superimposed images are shown in Figure S3

The heme active site is shown in Fig. 2B, and comprises the His 170 proximal ligand, and distal histidine (His 42) and arginine (Arg 38) residues. The distance between the heme iron and the Nε atom of the proximal His 170 is 2.0 Å, while that between the heme iron and Nε of distal histidine (His 42) is 6.1 Å. An ethylene glycol (EDO) situated at the δ-heme edge of the active site is at 4.0 Å from iron (Fe–O2). The electron densities of ethylene glycol and heme are shown in Fig. 2B. Ethylene glycol does not interact with the heme iron, but potentially forms hydrogen bonds with the distal histidine (O2-Nε = 2.7 Å) and arginine (O2-Nε = 3.1 Å, O1-Nη = 3.0 Å) residues (Fig. 2C). There is a residual density in the *F*_*o*_*-F*_*c*_ map near the ethylene glycol corresponding to a molecule of triethylene glycol (a possible contaminant of PEG), but cannot be interpreted with confidence (Figure S2). There is no distal water molecule within the bonding distance of the iron on the distal side (Fig. 3A), and there is no ferulic acid binding at the δ-heme edge (as has been observed in previous HRP structures, Fig. 3B and Figure S3A). The presence of ethylene glycol at the δ-heme edge—close to where other substrates [21], including benzhydroxamic acid in HRP (Fig. 3C and Figure S3B) [22], are known to bind—begs the question as to whether ferulic acid has left the pocket during crystallization, as a consequence of the new crystallization conditions used in this work (pH 8.5 here, compared to pH 6.5 in previous work [7], see Table S2), or whether it was replaced by ethylene glycol during the cryoprotection process in which crystals were soaked in ethylene glycol.

## Discussion

Horseradish peroxidase has been studied for more than two centuries, ever since Planche identified a colour change when fresh horseradish roots were mixed with guaiacol tincture [1]. The HRP enzyme, with its colourful redox intermediates of different metal oxidation states, was used in the early development of the stopped-flow method [23]. Dozens of other peroxidases were later identified, although none have attracted nearly as much attention as HRP. But despite its distinguished place at the top of the heme peroxidase hierarchy, very few crystal structures of HRP have ever appeared, and those that have were mainly published more than 20 years ago (Table S2). The difficulty of expressing the heavily glycosylated HRP enzyme is largely to blame for this lack of structural information when compared with other peroxidases (by comparison, there are more than 200 structures of cytochrome *c* peroxidase in the PDB). Since the original expression system for HRP was published [4], a number of other expression systems to produce recombinant HRP have appeared in the literature [24–33]. Only the original *E. coli* system [4] led to successful crystallization of the enzyme, but the yields were very low from this system. This, along with the limited range of crystallization conditions that have been explored, accounts for the lack of structural work on HRP.

In this work, we have obtained crystals from HRP produced recombinantly using a recently published production process [5]. All conditions used the substrate ferulic acid in the crystallization. Ferulic acid is well established as a substrate for HRP, and its binding location has been examined previously [7]. Oxidation of ferulic acid by HRP is faster than for some other aromatic acid substrates [7], which may be linked to cell wall biosynthesis in plants [34, 35]. For many decades, the binding locations of these peroxidase–substrate interactions were unknown, but are now structurally well defined [21] (so much so that in some cases, for example ascorbate peroxidase, cellular peroxidase activity assays can be used for proteomic applications [36]). Although ferulic acid was used as an additive in the crystallization, the protein crystal was devoid of any ferulic acid in the active site of ferric-HRP. Instead, ethylene glycol was observed in an equivalent location. It was reported [7] that the electron density maps for the ferulic acid ligand in the HRP–ferulic acid complex had a high degree of disorder in the crystal, suggesting weak interaction with HRP. Hence, multiple steps of crystallization may have led to the removal of ferulic acid from the HRP active site, which was occupied by ethylene glycol during cryoprotection.

The successful crystallization of HRP under a much wider range of conditions (Table S2) now opens opportunities to optimize the size/morphology of the crystals to suit the experimental requirements. We envisage this being useful for the production of microcrystals for XFEL work, or large crystals for neutron diffraction experiments in the future. This, in turn, would open up the possibility of exploring the nature and reactivity of the iconic Compound I and Compound II reaction intermediates of HRP.

## Supplementary Information

Below is the link to the electronic supplementary material.

Supplementary file1 (DOCX 1504 KB)

## Data Availability

The crystallographic data for the structures discussed in this article have been deposited at the wwPDB protein data bank under the deposition PDB ID 9H1M. The X-ray structure validation report has been submitted in the related files section. All other relevant data generated and analyzed during this study, including experimental, spectroscopic, and crystallographic data, are included in this article and its supplementary information.

## Abbreviations

HRP: Horseradish peroxidase
*E. coli*: *Escherichia coli*
SPG: Succinic acid, sodium hydrogen phosphate, glycine
MX: Macromolecular crystallography
autoPROC: Software for automatic processing of X-Ray diffraction data
XDS: X-ray Detector Software
CCP4: Collaborative Computational Project Number 4
UV: Ultraviolet
EDO: Ethylene glycol
XFEL: X-Ray free electron laser
FA: Ferulic acid
