# Supplementary material for: Crystal structure of ferric recombinant horseradish peroxidase
Source: J Biol Inorg Chem. 2025 Mar 7;30(3):221–7. doi: 10.1007/s00775-025-02103-2 (PMC11965164; doi:10.1007/s00775-025-02103-2)

**Crystal structure of recombinant ferric horseradish peroxidase**

Mst Luthfun Nesa,^1^ Suman K. Mandal,^1^ Christine Toelzer,^2^ Diana Humer,^3^ Peter C. E. Moody,^4^ Imre Berger,^2^ Oliver Spadiut,^3^ and Emma L. Raven^1^

*^1^ School of Chemistry, University of Bristol, UK*

*^2^ School of Biochemistry, University of Bristol, UK*

*^3^ Institute of Chemical, Environmental and Bioscience Engineering, Research Division Biochemical Engineering, TU Vienna, Austria*

*Leicester Institute for Structural & Chemical Biology, Department Molecular & Cell Biology, Henry Wellcome Building, University of Leicester, Leicester, LE1 7RH, UK*

**Figure S1**. SDS-PAGE analysis of purified recombinant HRP, showing a band consistent with the molecular weight of 34.05 kDa (12% acrylamide gel, molecular weight markers 10 to 250 kDa, New England Biolab - P7719S).


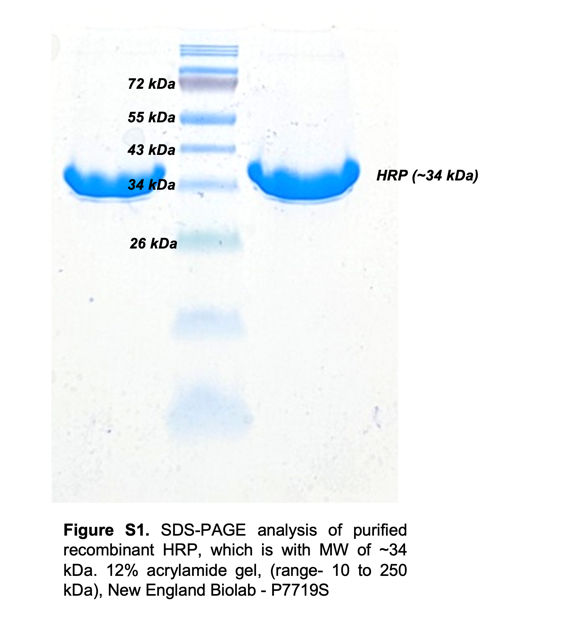


**Figure S2.** Triethylene glycol fits into the green density (2.5σl *F_o_-F_c_* map) next to the ethylene glycol (EDO) at the active site.

**
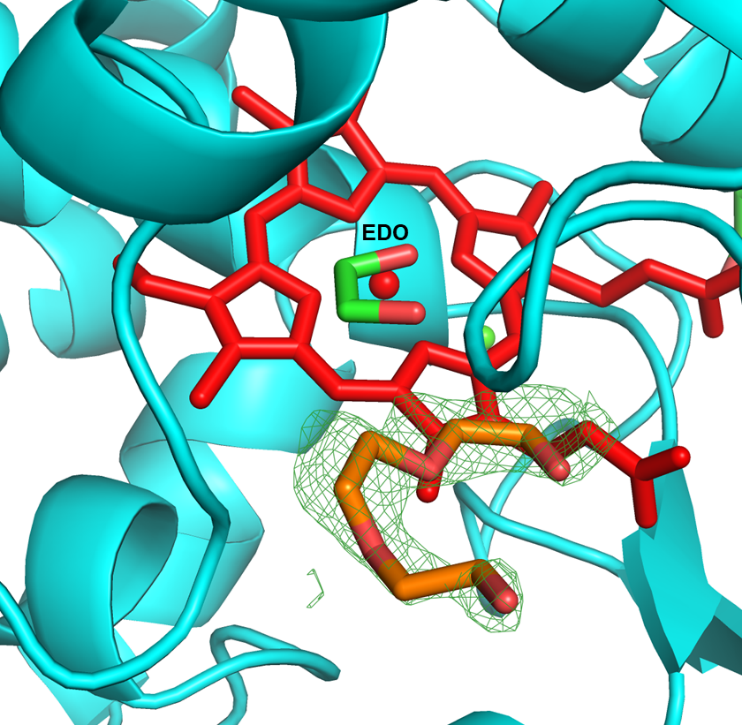
**

**Table S1**. List of crystallisation hits under different conditions. In all cases, ferulic acid was added to the crystallisation drop (see Methods).

| **Conditions** | **Composition of crystallisation solution** | **Images** |
| --- | --- | --- |
| **1** | 35% (v/v) MPD, 100 mM Imidazole/ Hydrochloric acid, pH 8.0, 200 mM Magnesium chloride | **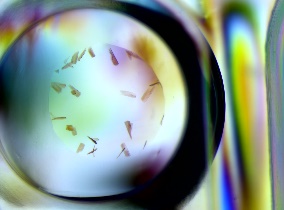** |
| **2** | 35% (v/v)~~,~~ MPD, 100 mM Imidazole/ Hydrochloric acid, pH 8.0, 200 mM Magnesium chloride | **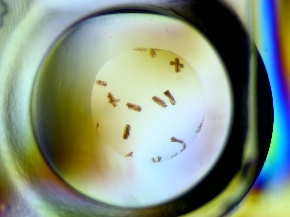** |
| **3** | 30% (w/v) PEG 8000, 100 mM Sodium acetate/ Acetic acid, pH 4.5, 200 mM Lithium sulfate | **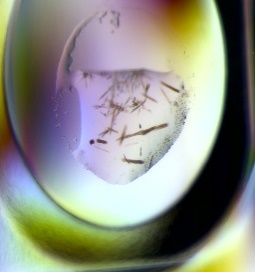** |
| **4** | 30% (w/v) PEG 8000, 100 mM Sodium acetate/ Acetic acid, pH 4.5, 200 mM Lithium sulfate | **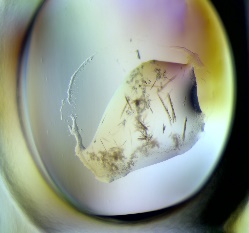** |
| **5** | 20% (w/v) PEG 1000, 100 mM Sodium phosphate dibasic/ Citric acid, pH 4.2, 200 mM Lithium sulfate | **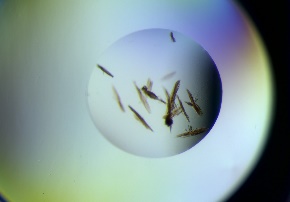** |
| **6** | 20% (w/v) PEG 1000, 100 mM Sodium phosphate dibasic/ Citric acid, pH 4.2, 200 mM Lithium sulfate | **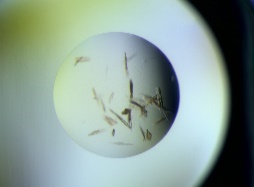** |
| **7** | 30% (w/v) PEG 3000, 100 mM CHES/ Sodium hydroxide, pH 9.5 | **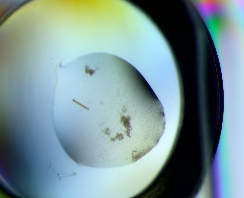** |
| **8** | 20% (w/v) PEG 1000, 100 mM Potassium phosphate monobasic/ Sodium phosphate dibasic, pH 6.2, 200 mM Sodium chloride | **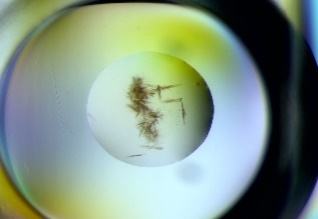** |
| **9** | 10% (w/v) PEG 8000, 100 mM Potassium phosphate monobasic/ Sodium phosphate dibasic, pH 6.2, 200 mM Sodium chloride | **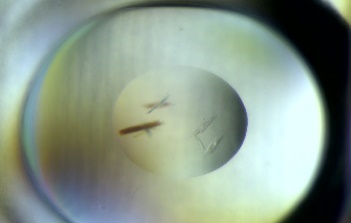** |
| **10** | 30% (v/v) MPD, 100 mM Sodium acetate/ Hydrochloric acid, pH 4.6, 20 mM Calcium chloride | **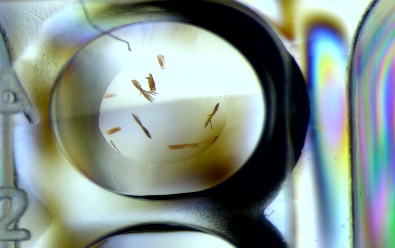** |
| **11** | 30% (v/v) MPD, 100 mM Sodium acetate/ Hydrochloric acid, pH 4.6, 20 mM Calcium chloride | **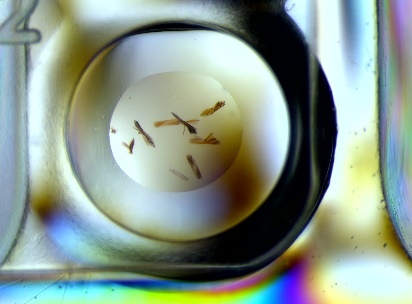** |
| **12** | 20% (w/v) PEG 3350, 200 mM Magnesium formate | **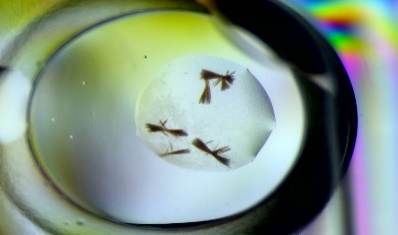** |
| **13** | 20% (w/v) PEG 6000, 100 mM Citric acid/ Sodium hydroxide, pH 4.0, 1000 mM Lithium chloride | **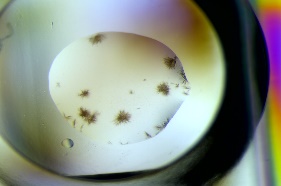** |
| **14** | 25% (w/v) PEG 1500, 100 mM SPG buffer, pH 6.5 | **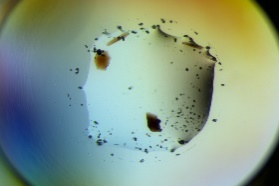** |
| **15** | 25% (w/v) PEG 1500, 100 mM SPG buffer, pH 6.5 | **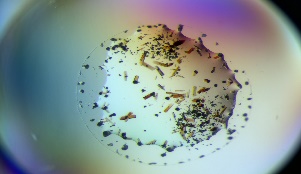** |
| **16** | 25% (w/v) PEG 1500, 100 mM SPG buffer, pH 8.5 | **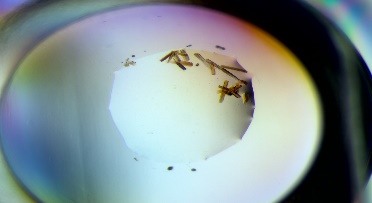** |
| **17** | 25% (w/v) PEG 1500, 100 mM SPG buffer, pH 8.5 | **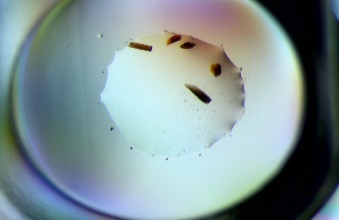** |
| **18** | 25% (w/v) PEG 1500, 100 mM MMT buffer, pH 6.5 | **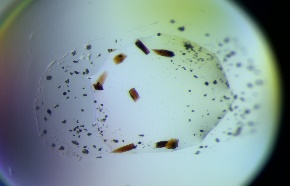** |
| **19** | 25% (w/v) PEG 1500, 100 mM MMT buffer, pH 6.5 | **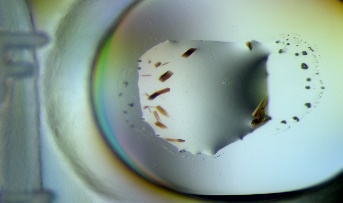** |
| **20** | 25% (w/v) PEG 1500, 100 mM MMT buffer, pH 9.0 | **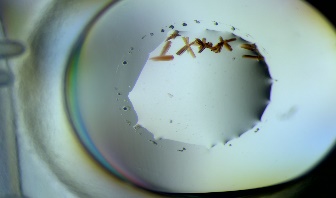** |
| **21** | 30% (w/v) PEG 2000~~,~~ MME, 150 mM Potassium bromide | **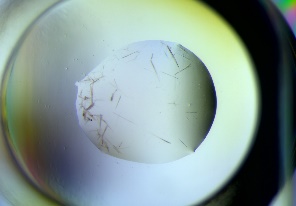** |
| **22** | 30% (w/v) PEG 2000, MME, 150 mM Potassium bromide | **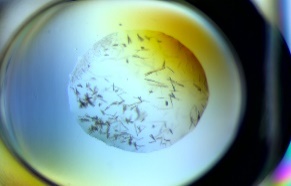** |
| **23** | 10% (w/v) PEG 2000~~,~~ MME, 100 mM Sodium acetate/ Acetic acid, pH 5.5, 200 mM Ammonium sulfate | **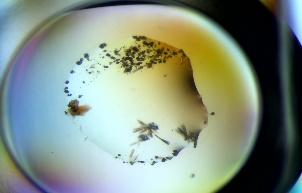** |
| **24** | 20% (w/v) PEG 2000~~,~~ MME, 100 mM Tris base/ Hydrochloric acid, pH 8.5, 200 mM Trimethylamine n-oxide | **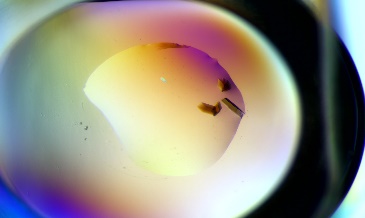** |
| **25** | 40% (v/v) 2-propanol, 100 mM Imidazole/ Hydrochloric acid, pH 6.5, 15% (w/v) PEG 8000 | **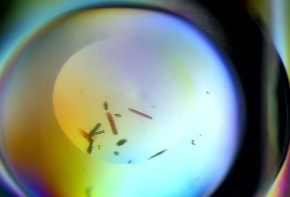** |
| **26** | 17% (w/v) PEG 10,000, 100 mM Bis tris/ Hydrochloric acid, pH 5.5, 100 mM Ammonium acetate | **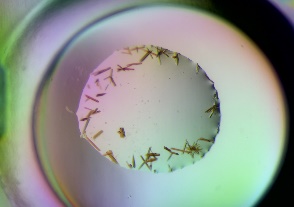** |

**Table S2.** Structures of HRP and various mutants available in the PDB.

| **PDB** | **Release date** | **Type** | **Space group** | **Resolution (Å)** | **Crystallization condition** | **Reference** |
| --- | --- | --- | --- | --- | --- | --- |
| 2ATJ | 28/1/98 | Wild type | *P 1 2_1_ 1* | 2 | 1.0 M Ammonium Dihydrogen Phosphate, 0.1 M Na-Cacodylate pH 6.5, Benzhydroxamic Acid as additive, Hanging Drop, 283 K | (1) |
| 1ATJ^a^ | 04/2/98 | Wild type | *P 3_1_ 1 2* | 2.15 | 16% (W/V) PEG 4000, 0.2 M Zinc Acetate and 0.1 M Cacodylate Buffer, pH 6.5 | (2) |
| 3ATJ | 28/4/99 | F221M | *P 1 2_1_ 1* | 2.2 | N/A | Unpublished |
| 6ATJ | 14/1/00 | Wild type | *P 2_1_ 2_1_ 2_1_* | 2 | 16% (W/V) PEG 4000, 0.2 M Calcium Acetate And 0.1 M Cacodylate Buffer, Ferulic Acid as additive, pH 6.5 | (3) |
| 7ATJ | 14/1/00 |  |  | 1.47 |  |  |
| 1H5D | 27/5/02 | Wild type | *P 2_1_ 2_1_ 2_1_* | 1.6 | 20% (W/V) PEG 4000, 0.2 M Calcium Acetate, 0.1 M Cacodylate Buffer, Ferulic Acid as additive, pH 6.5 | (4) |
| 1H5E | 27/5/02 |  |  | 1.6 |  |  |
| 1H5F | 27/5/02 |  |  | 1.6 |  |  |
| 1H5G | 27/5/02 |  |  | 1.6 |  |  |
| 1H5I | 27/5/02 |  |  | 1.6 |  |  |
| 1H5J | 27/5/02 |  |  | 1.6 |  |  |
| 1H5K | 27/5/02 |  |  | 1.6 |  |  |
| 1H5M | 27/5/02 |  |  | 1.6 |  |  |
| 1H57 | 17/6/02 |  |  | 1.6 |  |  |
| 1H55 | 18/6/02 |  |  | 1.61 |  |  |
| 1H5A | 18/6/02 |  |  | 1.6 |  |  |
| 1H5C | 18/6/02 |  |  | 1.62 |  |  |
| 1H58 | 18/6/02 |  |  | 1.7 |  |  |
| 1H5H | 21/6/02 |  |  | 1.6 |  |  |
| 1H5L | 21/6/02 |  |  | 1.6 |  |  |
| 1HCH | 19/7/02 |  |  | 1.57 |  |  |
| 4ATJ | 02/10/02 | H42E | *P 1 2_1_ 1* | 2.5 | 20% PEG 8000, 1.2 M Ammonium Dihydrogen Phosphate, 0.1 M Na-Cacodylate pH 6.5, Benzhydroxamic Acid as Additive, Hanging Drop, 283 K | (5) |
| 1KZM | 09/10/02 | R38S/H42E | *P 2_1_ 2_1_ 2_1_* | 2 | 20% PEG 8000, 0.2 M Ca-Acetate, 0.1 M Na-Cacodylate pH 6.5, Ferulic Acid as Additive, Hanging Drop, 283 K |  |
| 1GWU | 28/03/03 | A140G | *P 2_1_ 2_1_ 2_1_* | 1.31 | 20% (W/V) PEG 4000, 0.2 M Calcium Acetate, 0.1 M Cacodylate Buffer, pH 6.5 | Unpublished |
| 1GWO | 28/03/03 | A170Q |  | 2.07 |  |  |
| 1GWT | 28/03/03 | F221M |  | 1.7 |  |  |
| 1GW2 | 28/03/03 | T171S |  | 2.15 |  |  |
| 1GX2 | 28/03/03 | F209S | *P 1 2_1_ 1* | 2.2 |  |  |
| 1W4Y | 19/01/05 | Wild type | *P 2_1_ 2_1_ 2_1_* | 1.6 | 20% PEG 8000, 0.2 M Ca-Acetate, 0.1 M Na-Cacodylate pH 6.5, Ferulic Acid as additive, Hanging Drop, Streak Seeding, 277 K | (6) |
| 1W4W | 19/01/05 |  |  | 1.55 |  |  |
| 2YLJ | 13/06/12 | S167Y | *P 2_1_ 2_1_ 2_1_* | 1.69 | N/A | (7) |

^a^ PDB ID 1ATJ is the only crystal structure where ferulic acid/benzhydroxamic acid is not used as an additive and is the only crystal with a P 3_1_ 1 2 space group.

1. Henriksen, A., Schuller, D. J., Meno, K., Welinder, K. G., Smith, A. T., and Gajhede, M. (1998) Structural interactions between horseradish peroxidase C and the substrate benzhydroxamic acid determined by X-ray crystallography. *Biochemistry* **37**, 8054-8060

2. Gajhede, M., Schuller, D. J., Henriksen, A., Smith, A. T., and Poulos, T. L. (1997) Crystal structure of horseradish peroxidase C at 2.15 ~~A~~ **Å** resolution. *Nature structural biology* **4**, 1032-1038

3. Henriksen, A., Smith, A. T., and Gajhede, M. (1999) The structures of the horseradish peroxidase C-ferulic acid complex and the ternary complex with cyanide suggest how peroxidases oxidize small phenolic substrates. *Journal of Biological Chemistry* **274**, 35005-35011

4. Berglund, G. I., Carlsson, G. H., Smith, A. T., Szoke, H., Henriksen, A., and Hajdu, J. (2002) The catalytic pathway of horseradish peroxidase at high resolution. *Nature* **417**, 463-468

5. Meno, K., Jennings, S., Smith, A. T., Henriksen, A., and Gajhede, M. (2002) Structural analysis of the two horseradish peroxidase catalytic residue variants H42E and R38S/H42E: implications for the catalytic cycle. *Acta Crystallogr D* **58**, 1803-1812

6. Carlsson, G. H., Nicholls, P., Svistunenko, D., Berglund, G. I., and Hajdu, J. (2005) Complexes of horseradish peroxidase with formate, acetate, and carbon monoxide. *Biochemistry* **44**, 635-642

7. Al-Fartusie, F. S. (2012) *PhD Thesis*

**Figure S3.** Superimposed image of the active site of HRP-EDO (PDB 9H1M) with (A) the HRP-FA complex (PDB 6ATJ, in pale blue) and (B) the HRP-BHA complex (PDB 2ATJ, in grey), respectively. Colour scheme showing the heme in red, Arg 38 and His 42 in magenta, and ethylene glycol in green for HRP with EDO.


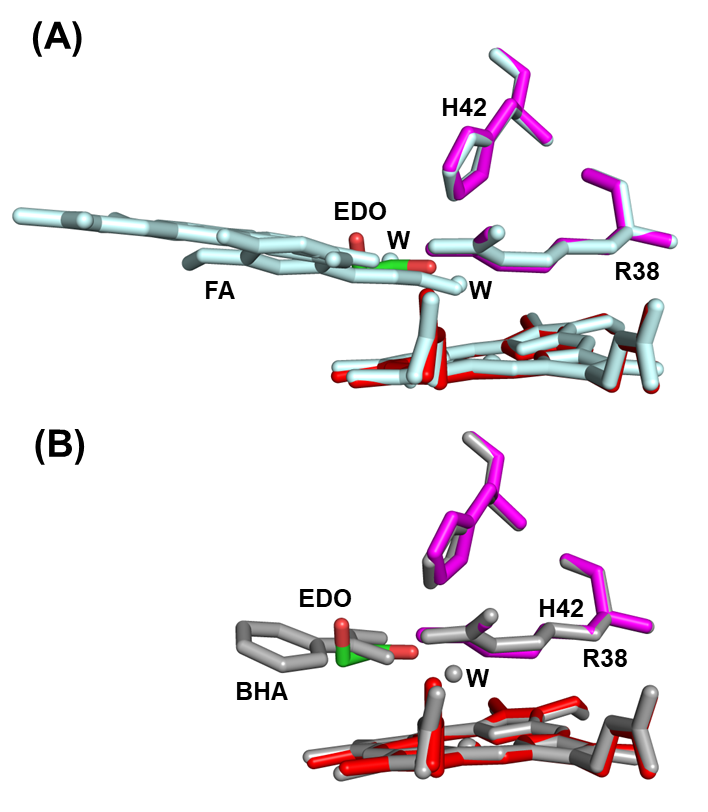

Supplement: Supplementary file 1 — Supplementary file1 (DOCX 1504 KB) [file 775_2025_2103_MOESM1_ESM.docx]
